# Pre-lymphodepletion & infusion endothelial activation and stress index as predictors of clinical outcomes in CAR-T therapy for B-cell lymphoma

**DOI:** 10.1038/s41408-022-00777-4

**Published:** 2023-01-05

**Authors:** Aldo A. Acosta-Medina, Isla McKerrow Johnson, Radhika Bansal, Matthew Hathcock, Saad J. Kenderian, Urshila Durani, Arushi Khurana, Yucai Wang, Jonas Paludo, Jose C. Villasboas, N. Nora Bennani, Patrick B. Johnston, Stephen M. Ansell, Yi Lin, Hassan B. Alkhateeb

**Affiliations:** 1 Department of Internal Medicine, Mayo Clinic, Rochester, MN USA; 2 Division of Hematology, Mayo Clinic, Rochester, MN USA

**Keywords:** Epidemiology, B-cell lymphoma

**TO THE EDITOR:**

Chimeric antigen receptor T cell (CAR-T) therapy has been standard of care for multiple hematologic malignancies in the past few years. However, CAR-T-related toxicities remain a significant barrier limiting its efficacy [1].

Endothelial dysfunction is known to play a pivotal role in the pathogenesis of life-threatening complications observed in patients undergoing bone marrow transplant [2]. The use of the Endothelial Activation and Stress Index (EASIX) score, as a proxy of systemic endothelial activation, has been validated for prediction of clinical outcomes after allogeneic stem cell transplantation [3]. There is growing evidence suggesting similar effects of aberrant endothelial activation partake in the development of both cytokine-release syndrome (CRS) and immune effector cell-associated neurotoxicity syndrome (ICANS) after CAR-T cell infusion [4, 5].

Pennisi et al. have recently published their CAR-T center experience in which increasing EASIX and modified EASIX (mEASIX) scores in the pre-lymphodepletion (pLD) setting were associated with severe CRS and ICANS [6]. Nevertheless, use of these scores in this clinical setting remains limited and the ideal time point to optimize their calculation remains unknown. Our aim was to compare the predictive capacity of the EASIX and mEASIX calculated pLD to scores at time of CAR-T infusion, in terms of CRS and ICANS.

A retrospective review including all consecutive patients ≥18 years-old undergoing CAR-T therapy with axicabtagene ciloleucel for B-cell lymphoma at Mayo Clinic from June 2016 to December 2020 was performed. Total accrual and sample size were entirely dependent on patients meeting inclusion criteria throughout the study period. Demographic, laboratory, and clinical data were abstracted from electronic medical records.

Both the Endothelial Activation and Stress Index (EASIX) score and the modified EASIX score (mEASIX) were calculated as per original publications [6, 7] by use of serum lactate dehydrogenase, platelet count, and either serum creatinine or C-reactive protein, respectively. Both scores were calculated at two distinct time points: infusion laboratory results were defined as those obtained from peripheral blood draw on the calendar day of CAR-T product administration prior to product infusion (day 0), while pre-lymphodepletion (pLD) results were defined as those prior to initiation of lymphodepleting chemotherapy on day −5 and up to day −15.

Cytokine release syndrome (CRS) and immune-effector cell-associated neurotoxicity syndrome, as well as their grading and resolution, were defined per the American Society for Transplantation and Cellular Therapy consensus guidelines [8]. Tocilizumab and steroid administration for management of complications were administered based on institution protocol. The study was deemed exempt by our Institutional Review Board and performed in accordance with the Declaration of Helsinki.

A total of 84 patients were included in our study, 65.5% (*n* = 55) of whom were male and 64.3% (*n* = 54) of whom had a baseline diagnosis of diffuse large B-cell lymphoma (Table 1). After CAR-T infusion, 83.3% (*n* = 70) of patients developed CRS, 2 of which were grade (G) 3-4. ICANS was observed in 52.4% (*n* = 44) of the cohort, 29.5% (*n* = 13) were G3-4. Overall, 29.8% of patients (*n* = 25) required use of tocilizumab for CRS management; 20% (*n* = 5) required >1 dose. A total of 29 patients (34.5%) required steroid administration for CRS/ICANS management, requiring a median cumulative dexamethasone dose of 110 mg (*range* 10–1100 mg). Of the cohort, 91.7% (*n* = 77) were alive and had an evaluable response at day +30. Best response to CAR-T product was a complete or partial response in 51.2% (*n* = 43) and 19% (*n* = 16) of patients, respectively.

**Table 1:** Baseline patient characteristics.

	*N* = 84
Age at CAR-T, *median* (range)	60 (19–74)
Male sex, *n* (%)	55 (65.5)
Baseline diagnosis, *n* (%)	
Diffuse large B-cell lymphoma	54 (64.3)
Transformed follicular lymphoma	21 (25.0)
High-grade B-cell lymphoma	8 (9.5)
Primary mediastinal B-cell lymphoma	1 (1.2)
*Pre-Lymphodepletion data*	
Serum laboratories^a^, *median* (range)	
Creatinine [mg/dL]	0.875 (0.48–2.24
Platelet [×10^9^/L]	157 (26–491)
Lactate dehydrogenase [U/L]	238 (107–4059)
Ferritin [mcg/L]	322 (17–11420)
C-Reactive protein [mg/L]	11.65 (2.9–231.7)
EASIX^b^, *median* (range)	1.783 (0.23–22.2)
mEASIX^b^, *median* (range)	2.567 (0.16–248.6)
*Infusion data*	
Serum laboratories^a^, *median* (range)	
Creatinine [mg/dL]	0.785 (0.41–2.64)
Platelet [×10^9^/L]	121.5 (2.9–251.7)
Lactate dehydrogenase [U/L]	233 (126–3347)
Ferritin [mcg/L]	573 (72–12980)
C-Reactive protein [mg/L]	17.65 (2.9–251.7)
EASIX^b^, *median* (range)	1.668 (0.29–27.5)
mEASIX^b^, *median* (range)	4.032 (0.25–458.3)

^a^Lactate dehydrogenase and ferritin data available in *n* = 83 and *n* = 81 patients.

^b^82 patients included.

## EASIX score evaluation

Complete laboratory evaluation enabled calculation of pLD- and I-EASIX in 82 patients. Median pLD-EASIX score was 1.78 (*range* 0.23–22.2). A non-significant decrease in score to 1.67 (*range* 0.29–27.5) at the time of CAR-T infusion (*p* = 0.18) was observed. Low incidence of G3-4 CRS precluded analysis of interactions. There was no significant association between duration of CRS and the pLD-EASIX (*p* = 0.5) or I-EASIX (*p* = 0.7) scores. Increased risk of G3-4 ICANS was observed with higher pLD-EASIX (OR 1.1, 95%CI 1.002–1.285; *p* = 0.04) and I-EASIX (OR 1.19, 95%CI 1.047–1.357; *p* = 0.008). No difference between duration of ICANS with higher I-EASIX (*p* = 0.09) or pLD-EASIX (*p* = 0.74) was present.

In terms of treatment, neither pLD nor I-EASIX were associated with increased tocilizumab requirements. Increasing pLD-EASIX (R^2^ = 0.066; *p* = 0.019) and I-EASIX (R^2^ = 0.132; *p* = 0.001) were both associated with increased cumulative steroid dosing for CRS/ICANS management.

## Modified EASIX score evaluation

Complete laboratory evaluation enabled calculation of pLD- and I-mEASIX in 82 patients. Median pLD-mEASIX score was 2.57 (*range* 0.16–248.5) with an observed significant increase to 4.03 (*range* 0.25–458.1) at time of CAR-T infusion (*p* < 0.001). I-mEASIX was significantly associated with an increased risk of G3-4 ICANS (OR 1.008, 95% CI 1.001–1.015; *p* = 0.034) and with increased cumulative steroid dosing (R^2^ = 0.080; *p* = 0.010). No additional associations were observed during evaluation of mEASIX with other clinical outcomes (Table 2).

**Table 2:** Analysis of correlation between EASIX, CRP, and mEASIX and clinical outcomes.

	ORR	CRS	ICANS	ICANS G3-4	Tocilizumab requirement	Cumulative steroid dose	ROC curve analysis
EASIX	[OR; *p*]	[OR; *p*]	[OR; *p*]	[OR; *p*]	[OR; *p*] [OR; *p*]	[β; R^2^; *p*]	[AUC; *p*]
Pre-lymphodepletion	0.980; *0.747*	1.166; *0.296*	1.015; *0.790*	1.135; ***0.047***	1.030; *0.622*	14.22; 0.066; ***0.019***	0.571; *0.42*
Infusion	0.939; *0.238*	1.129; *0.299*	1.066; *0.267*	1.192; ***0.008***	1.071; *0.196*	17.75; 0.132; ***0.001***	0.623; *0.161*
C-reactive protein	[OR; *p*]	[OR; *p*]	[OR; *p*]	[OR; *p*]	[OR; *p*]	[β; R^2^; *p*]	
Pre-lymphodepletion	0.878; ***0.007***	1.102; *0.256*	1.081; *0.098*	1.045; *0.342*	1.114; ***0.015***	5.759; 0.022; *0.179*	–
Infusion	0.915; *0.50*	1.060; *0.402*	1.093; *0.070*	1.107; ***0.031***	1.122; ***0.013***	8.440; 0.046; *0.054*	
Modified EASIX	[OR; *p*]	[OR; *p*]	[OR; *p*]	[OR; *p*]	[OR; *p*]	[β; R^2^; *p*]	[AUC; *p*]
Pre-lymphodepletion	0.993; *0.184*	1.100; *0.229*	1.007; *0.306*	1.007; *0.242*	1.007; *0.205*	1.043; 0.042; *0.066*	–
Infusion	0.995; *0.155*	1.056; *0.262*	1.011; *0.119*	1.008; ***0.034***	1.007; *0.086*	0.847; 0.080; ***0.010***	0.716; ***0.014***
I-mEASIX, [OR; *p*]							
High (≥4) vs. Low (<4) risk		2.025; *0.240*	1.103; *0.825*	4.086; ***0.034***	2.051; *0.145*	1.002; *0.178*	2.025; *0.240*

EASIX = LDH * Creatinine/Thrombocytes; Modified EASIX = LDH * CRP/Thrombocytes.

β Beta coefficient & R-squared, rather than OR, reported for linear regression.

*AUC* area under the curve, *CRS* cytokine release syndrome, *ICANS* immune effector cell-associated neurotoxicity syndrome, *EASIX* Endothelial Activation and Stress Index, *OR* odds ratio, *ORR* overall response rate, *ROC* receiver operating characteristic.

Bold values indicates statistically significant.

Given the significant association between G3-4 ICANS and the I-EASIX and I-mEASIX scores, determination of ideal cutoff was performed via ROC analysis. Area under the curve (AUC) for the I-mEASIX (AUC = 0.716; *p* = 0.014), but not I-EASIX (*p* = 0.161), determined a statistically significant cutoff for severe ICANS prediction. An I-mEASIX of ≥4 (Sen = 76.9%, Sp = 55.1%) best predicted high-risk for G3-4 ICANS (OR 4.086, CI 1.033–16.16; *p* = 0.034).

In this external cohort, we were able to observe similar associations between the EASIX scores and unfavorable clinical outcomes as those reported in the available literature. Whilst associations between higher rates of ICANS were observed in patients with both higher EASIX and higher mEASIX scores, these were significantly optimized when calculated from laboratory workup obtained at time of CAR-T infusion as compared to those obtained prior to initiation of lymphodepletion. Particularly, both I-EASIX and I-mEASIX were associated with increased risk of G3-4 ICANS and increased cumulative dose of steroid.

Of note, only the mEASIX was able to categorically predict high and low-risk patients (cutoff ≥4), likely evidencing the impact of CRP as an inflammatory marker as opposed to creatinine, with higher CRP at time of infusion being associated with an increased risk of G3-4 ICANS (*p* = 0.031) as well as with decreased ORR (*p* = 0.05). Other inflammatory acute phase reactants evaluated in our sample population, including serum ferritin both at the time of infusion (*p* = 0.82) and pLD (*p* = 0.74), were not further pursued given no signal regarding its association with adverse outcomes. It is an important consideration that prior groups’ experience (Greenbaum *Blood Advances* 2021) has only demonstrated association of ferritin to CRS and ICANS in low-risk EASIX groups given the significant correlation between high-risk EASIX and high serum ferritin.

These results as well as the lack of association of EASIX with CRS in our cohort, differ from prior published experience. Possible explanations may include the limited rates of severe CRS (*n* = 2) in our cohort, the exclusive use of CD28 costimulatory-based cellular products in our patients, the inclusion of a limited population of transformed indolent B-cell lymphomas, in addition to sample size.

Nevertheless, our results provide additional information regarding the validity of endothelial activation indexes in predicting complications after CAR-T infusion. Additionally, our results suggest a possible association between increasing EASIX and increasing cumulative steroid requirement not priorly evidenced in the published literature. Though it is important to note that only a limited proportion of variability within steroid dosing in our cohort was predictable with EASIX.

It remains clear that further investigation of additional, more specific, markers of endothelial activation is called for in future studies.

### Statistical analysis

Nominal and ordinal variables were described in terms of frequency and percentage, while quantitative data were described in terms of median and range. Univariate association between the EASIX scoring systems and clinical outcomes was performed using classical logistic regression for categorical outcomes or with linear regression for continuous outcomes. Evaluation of differences in pLD-EASIX as compared to paired I-EASIX was performed via Wilcoxon’s signed-ranks test. All calculations were performed with IBM’s SPSS Statistics version 27.

## Data Availability

Data will be provided upon direct request to the authors. For original data request contact AcostaMedina.Aldo@mayo.edu and Alkhateeb.Hassan@mayo.edu.
